# Sphenoidal Meningoencephalocele Secondary to a Persistent Sternberg Canal: A Case Report

**DOI:** 10.7759/cureus.84332

**Published:** 2025-05-18

**Authors:** Anouar Ben Ameur El Youbi, Mariam Ameziane Hassani, Abdellatif Oudidi, Mohamed Nouredine El Amine El Alami

**Affiliations:** 1 Ear, Nose, and Throat Department, University Hospital Center Hassan II, Fes, MAR

**Keywords:** cerebrospinal fluid, lateral craniopharyngeal canal, meningoencephalocele, rhinorrhea, sternberg canal

## Abstract

The Sternberg canal represents a congenital defect of the lateral wall of the sphenoid sinus, predisposing to spontaneous cerebrospinal fluid (CSF) leakage and the formation of meningoencephaloceles. We report the case of a 54-year-old patient presenting with a four-month history of anterior rhinorrhea in the absence of any other associated symptoms. High-resolution computed tomography and magnetic resonance imaging identified an osteomeningeal defect in the lateral sphenoid sinus wall, associated with a meningoencephalocele. The patient underwent a right transsphenoidal sphenoidotomy with multilayer reconstruction utilizing abdominal fat, conchal cartilage, and biological adhesive. Favorable clinical outcomes were achieved, with no recurrence noted during a nine-month follow-up period. Surgical repair of CSF leaks through the Sternberg canal remains technically demanding, primarily due to the anatomical complexity and restricted accessibility of the lateral sphenoid recess.

## Introduction

The sphenoid sinus is an uncommon site for osteomeningeal defects, particularly those of spontaneous origin [1,2]. Recent investigations have implicated the Sternberg canal as a potential anatomical pathway predisposing to spontaneous cerebrospinal fluid (CSF) leaks into the sphenoid sinus [3,4]. The lateral craniopharyngeal canal, commonly called the Sternberg canal, represents a complex embryological structure resulting from incomplete ossification of the sphenoid bone during development [5]. We describe a case of intrasphenoidal meningoencephalocele secondary to the persistence of the lateral craniopharyngeal canal. The patient underwent a right trans-sphenoidal sphenoidotomy with closure of the sphenoid sinus defect. Postoperative follow-up demonstrated favorable clinical outcomes with complete resolution of symptoms.

## Case presentation

A 54-year-old patient with no notable medical history presented with persistent anterior rhinorrhea for four months, in the absence of other associated functional symptoms. On clinical examination, the patient appeared to be in good general health, with a blood pressure of 130/80 mmHg, a heart rate of 95 beats per minute, and a body mass index of 28 kg/m². Otorhinolaryngological evaluation revealed abundant, clear, and spontaneous rhinorrhea originating from the right nasal cavity, with the patient reporting a salty taste. The remainder of the clinical examination was unremarkable.

**Figure 1:**
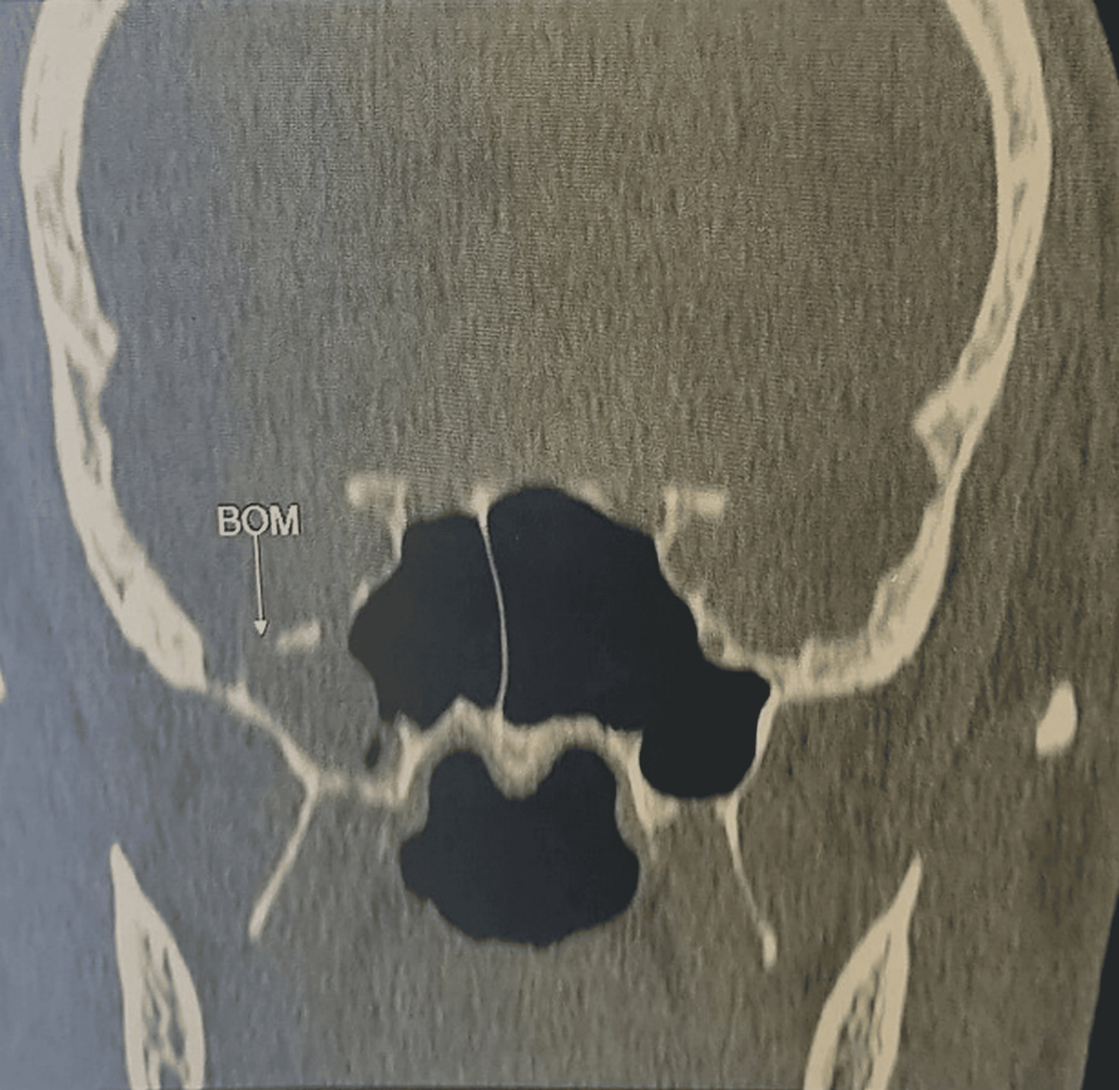
Axial CT scan showing a bony defect in the lateral wall of the sphenoid sinus with herniation of cerebro-meningeal tissue (arrow) CT: computed tomography

**Figure 2:**
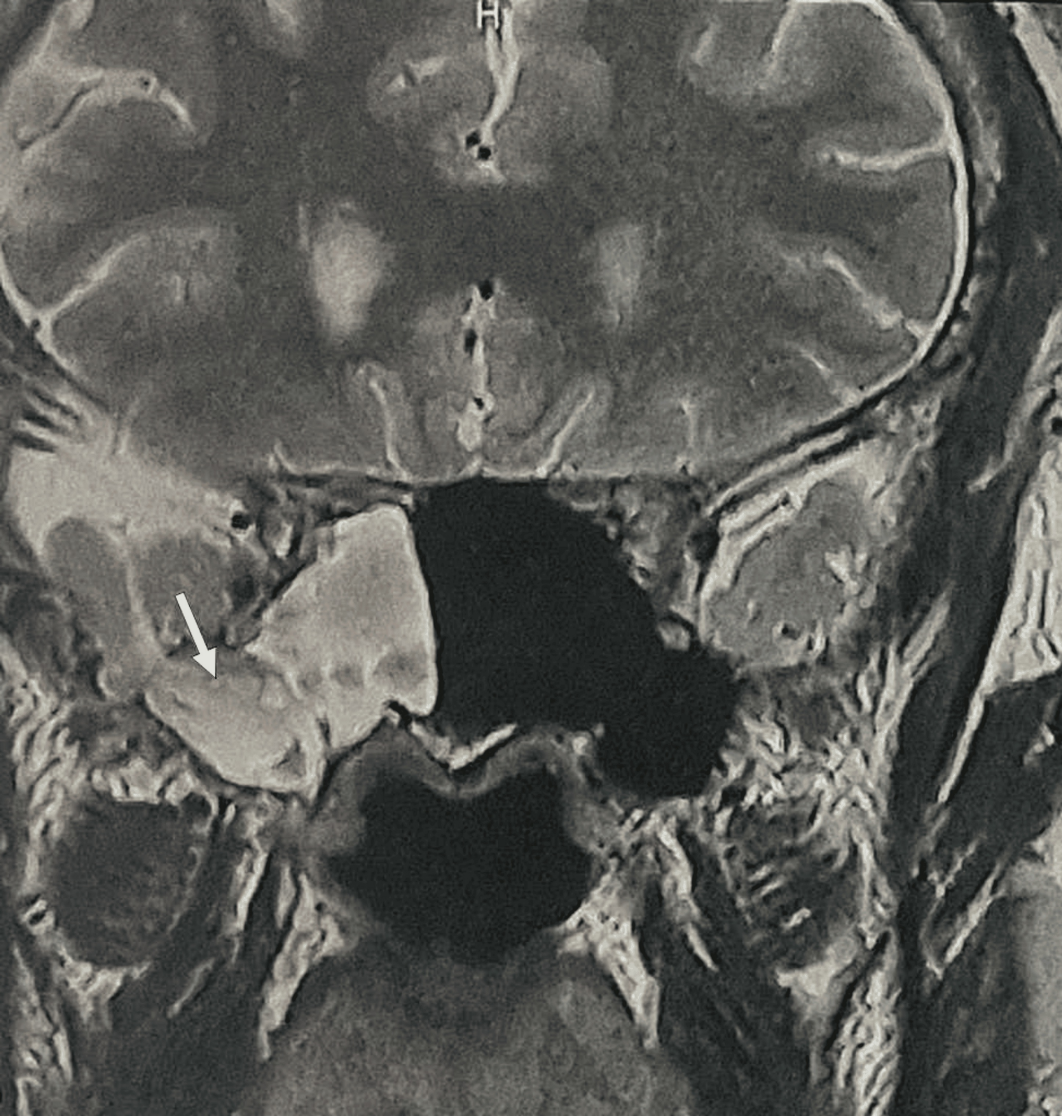
Coronal MRI demonstrating an osteomeningeal defect in the posterolateral wall of the sphenoid sinus associated with a meningoencephalocele (arrow) MRI: magnetic resonance imaging

A computed tomography (CT) scan demonstrated a bony defect measuring 8 × 4 mm in the lateral wall of the right sphenoid sinus, associated with herniation of brain parenchyma and meninges (Figure 1). Subsequent magnetic resonance imaging (MRI) confirmed the presence of an osteomeningeal defect in the posterolateral wall of the sphenoid sinus, associated with a meningoencephalocele (Figure 2).

**Figure 3:**
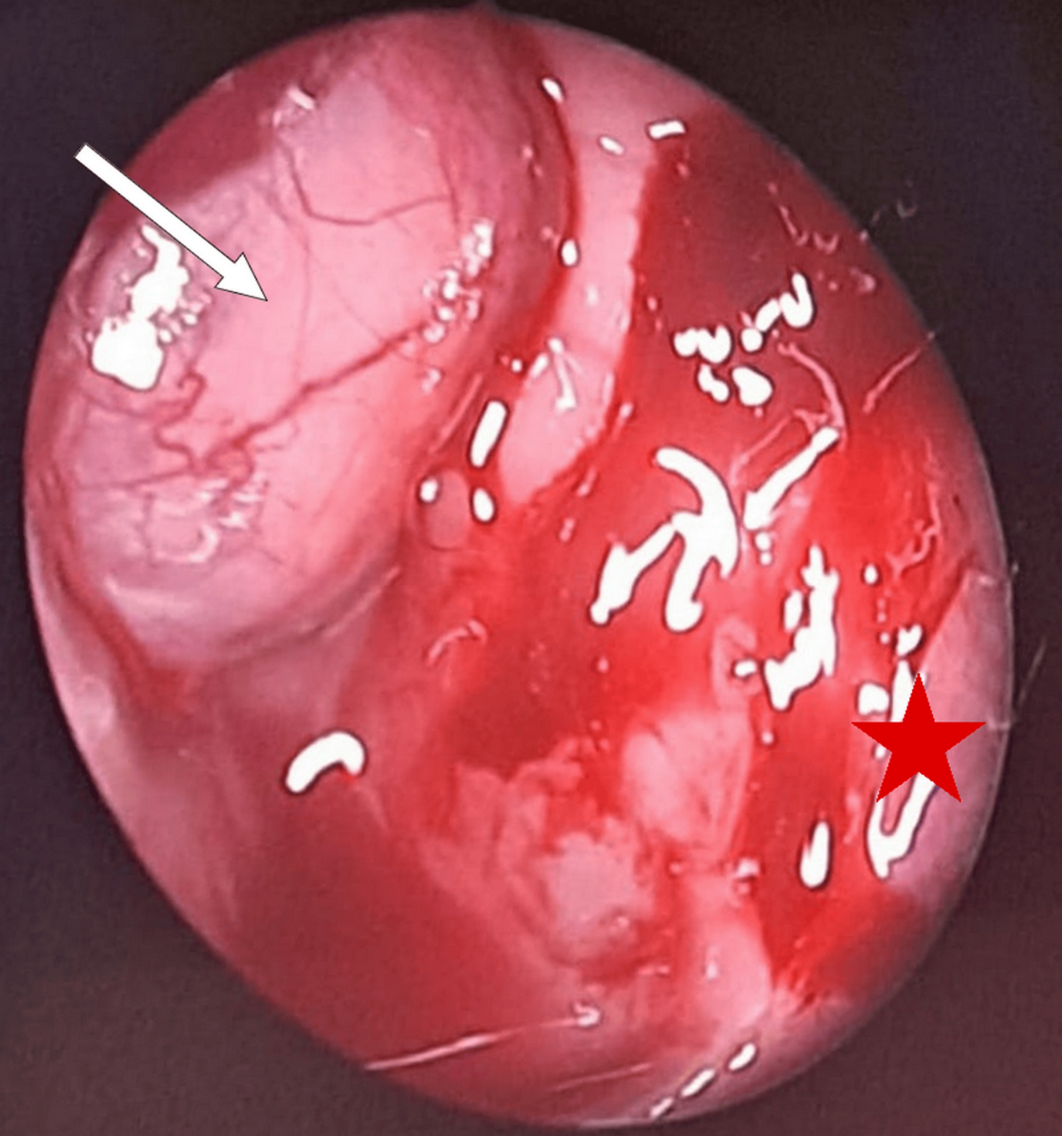
Endoscopic image showing a meningoencephalocele (arrow) protruding from the lateral wall of the sphenoid sinus (star)

**Figure 4:**
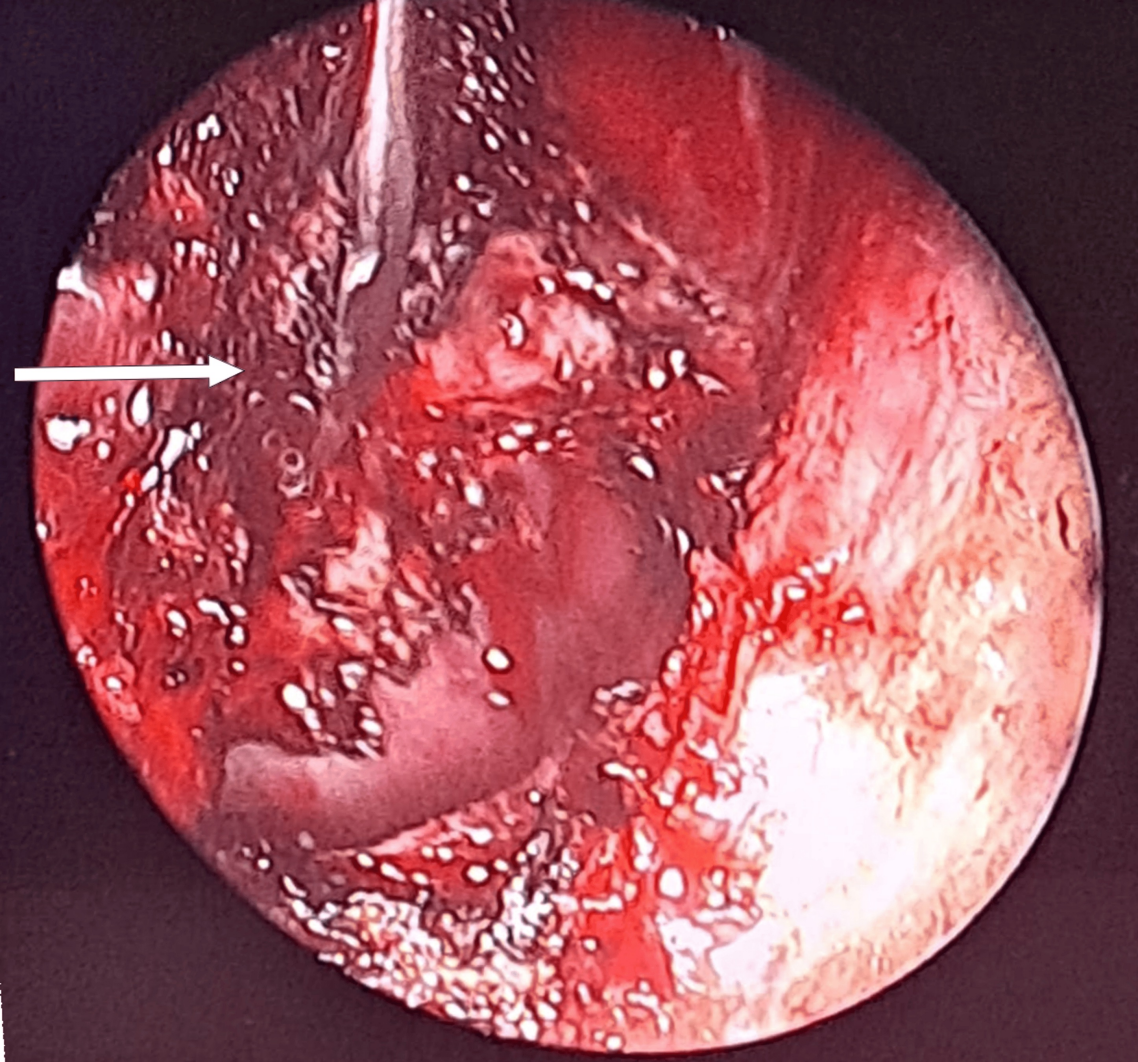
Endoscopic image showing the lateral wall of the sphenoid sinus after cauterization of the meningeal tissue (arrow)

The patient underwent a right trans-ostial sphenoidotomy under general anesthesia using an endoscopic approach, with enlargement of the sphenoid ostium. Intraoperative exploration of the sphenoid sinus revealed a meningoencephalocele protruding from the lateral wall of the sinus (Figure 3). Exclusion of the sphenoid sinus was performed, including cauterization of the meningeal component of the meningoencephalocele (Figure 4). Reconstruction was completed by sealing the sphenoid sinus with abdominal fat, conchal cartilage, and biological glue. Postoperative follow-up over nine months showed progressive improvement of clinical symptoms.

## Discussion

CSF rhinorrhea results from a discontinuity of the osteomeningeal barrier, allowing the leakage of CSF into pneumatized craniofacial cavities, particularly the paranasal sinuses [6,7]. Osteomeningeal defects are generally classified as secondary, related to trauma, surgical procedures, or tumors, or as spontaneous, arising in regions of inherent bony or meningeal weakness [6,8]. Spontaneous CSF leaks are relatively rare, accounting for only 3% to 4% of all cases, and predominantly involve the frontal and ethmoidal sinuses or the nasal cavity; the sphenoid sinus is an uncommon site [6]. Nevertheless, emerging data suggest that the sphenoid sinus may represent a distinct and underrecognized location for spontaneous CSF leaks, potentially exceeding secondary causes in this region [1,2].

This finding underscores the importance of considering specific anatomical predispositions. In particular, the persistence of the Sternberg canal, or lateral craniopharyngeal canal, has been proposed as a contributing factor [3,4]. Described by Cruveilhier in 1877 and Sternberg in 1888, this canal results from the incomplete fusion of the presphenoid and postsphenoid components during sphenoid bone development [4,5,9,10]. Its persistence in adulthood, reported in 0.1% to 4% of cases, may constitute a potential site of weakness facilitating spontaneous CSF leakage [5,9,11]. Anatomically, the Sternberg canal is situated in the posterolateral wall of the sphenoid sinus, inferolateral to the maxillary division (V2) of the trigeminal nerve [12].

Clinically, spontaneous CSF leaks typically manifest as persistent, watery rhinorrhea, exacerbated by forward head flexion and occasionally associated with posterior nasal drainage [12]. Radiological assessment is crucial for diagnosis. Sinus CT with multiplanar reconstructions enables the identification of bony defects and pneumocephalus. At the same time, MRI remains the preferred modality due to its multiplanar capabilities and superior sensitivity in detecting CSF fistulas, encephaloceles, and arachnoidoceles, particularly on T2-weighted sequences with thin sections [13].

Surgical repair of CSF leaks from the lateral recess of the sphenoid sinus remains technically demanding, primarily due to the depth and restricted access to this region [14]. The surgical approach must be tailored based on the extent of sphenoid sinus pneumatization, the size and location of the defect, and the ability to achieve a durable, watertight closure [10]. Endoscopic transnasal techniques are increasingly favored for their minimal invasiveness, avoidance of external scars, and reduced risk of temporal lobe manipulation [15]. Nevertheless, complications such as persistent leakage, neurovascular injury, and meningitis remain possible [14].

## Conclusions

Spontaneous CSF leaks of the sphenoid sinus, though rare, represent an increasingly recognized clinical entity with specific anatomical predispositions, notably the persistence of the Sternberg canal. Accurate diagnosis relies on high-resolution imaging, with MRI providing superior sensitivity for detecting associated osteomeningeal defects and encephaloceles. Given the lateral sphenoid recess's anatomical complexity and deep location, surgical management remains challenging and requires meticulous planning to achieve durable, watertight repairs. Endoscopic transnasal approaches offer a minimally invasive alternative, although potential complications such as persistent leakage and neurovascular injury must be carefully considered. Early diagnosis and appropriate surgical intervention are critical to prevent serious complications such as meningitis and to optimize patient outcomes.
